# Tomato Phenotypic Diversity Determined by Combined Approaches of Conventional and High-Throughput Tomato Analyzer Phenotyping

**DOI:** 10.3390/plants9020197

**Published:** 2020-02-05

**Authors:** Amol N. Nankar, Ivanka Tringovska, Stanislava Grozeva, Daniela Ganeva, Dimitrina Kostova

**Affiliations:** 1 Center of Plant Systems Biology and Biotechnology (CPSBB), 4000 Plovdiv, Bulgaria; dkostova2011@gmail.com or; 2 Maritsa Vegetable Crops Research Institute (MVCRI), 4003 Plovdiv, Bulgaria; dwdt@abv.bg (I.T.); stanislava_grozeva@abv.bg (S.G.); dganeva@abv.bg (D.G.)

**Keywords:** Tomato Analyzer, Bulgarian tomato, high-throughput fruit phenomics, data visualization, conventional phenotyping, fruit diversity, fruit morphometric and colorimetric traits, tomato genetic variability

## Abstract

Morphological variation in vegetative and fruit traits is a key determinant in unraveling phenotypic diversity. This study was designed to assess phenotypic diversity in tomatoes and examine intra- and intervarietal groups’ variability using 28 conventional descriptors (CDs) and 47 Tomato Analyzer (TA) descriptors related to plant and fruit morphometry. Comprehensive phenotyping of 150 accessions representing 21 countries discerned noticeable variability for CD vegetative traits and TA quantified fruit features, such as shape, size, and color. Hierarchical cluster analysis divided the accessions into 10 distinct classes based on fruit shape and size. Multivariate analysis was used to assess divergence in variable traits among populations. Eight principal components with an eigenvalue >1 were identified by factor analysis, which contributed 87.5% variation to the total cumulative variance with the first two components contributing 32.0% and 18.1% variance, respectively. The relationship between vegetative and fruit descriptors was explained by respective CD and TA correlation networks. There was a strong positive correlation between fruit shape and size whereas negative correlations were between fruit shape index, internal eccentricity, and proximal end shape. The combined approach of CD and TA phenotyping allowed us to unravel the phenotypic diversity of vegetative and reproductive trait variation evaluated at pre- and post-harvest stages.

## 1. Introduction

Tomato (*Solanum lycopersicum* L.) is an essential multipurpose vegetable, used in an array of fresh and processed foods. It is the second most important vegetable consumed and grown worldwide [1,2] due to its adaptability under different environments [3]. Tomatoes are rich in fiber, minerals, vitamins, carotenoids, and phenolic compounds [4,5], and are an enriched source of nutrients beneficial to human health [6,7]. Tomatoes are native of the South American Andes [8] and were brought to Europe at the beginning of the 16th century [9]. Despite its early introduction, tomatoes were not adapted as an edible crop until the early 18th century since they were mainly grown for ornamental purposes [10]. It was first adapted in the Mediterranean or European gardens of current-day Italy and Spain [10,11] followed by the subsequent introduction into the rest of Europe [12].

In the Balkans, tomatoes were successfully adapted and grown at the end of the 19th century [13]. For decades, landraces have been selected by farmers for fruit quality, subsistence, and their resilience under diverse environmental conditions [7,14,15]. Balkan tomatoes possess a unique flavor, taste, shape, and size that is very typical to this region, and are grown by farmers to retain the “Taste of the Past” [13,16,17]. Bulgaria is one of the Balkan countries with a long tradition of tomato growing and breeding. It was not accidental that here at Maritsa Vegetable Crops Research Institute (MVCRI) the world’s first tomato F_1_ hybrid was created in early 1934 [13]. Within years various local and introduced forms were introgressed to create a large number of breeding lines, F_1_ hybrids, and open pollinated varieties using conventional breeding [18,19,20]. However, there is no structured framework to describe and quantify tomato phenotypes existing in the current collection [20], hence the need for objective phenotypic evaluation is necessary.

Conventionally, morphological characterization is carried out using tomato descriptors [21] related to vegetative and reproductive traits. These descriptors are used to categorize tomato varietal groups [22,23,24,25] mostly based on fruit traits [26]. Characterization of fruit morphology is a classical approach for varietal identification and creation of varietal groups [27,28] and well suited for analysis of genetic diversity in regard to the effects of breeding and plant genetic resources conservation and utilization [29]. Application of high-throughput tools to characterize whole plant diversity in vegetables is still at the proof of concept stage [30], though they are invariably successful in most field crops [31]. Lack of affordable and accessible high-throughput tools appear to hinder the efficacy of most vegetable breeding programs. Overall, potential impediments to comprehensive fruit trait characterization are related to manual fruit measurements mostly used in conventional multi-location testing [32] that require intensive labor and time. However, a semi-automatic tool known as a Tomato Analyzer (TA) has proven highly efficient to characterize fruit morphometric [33] and colorimetric diversity [34] studied in an array of Solanaceous crops.

We hypothesized that inter- and intra-population plant and fruit diversity varies between varietal groups and within each varietal group. The goal of the present investigation is to understand phenotypic diversity in tomato accessions for the establishment of a tomato core collection. Towards this goal, intra- and intervarietal groups’ phenotypic diversity of tomato collections representing diverse geographic regions were measured and described using both conventional descriptors (CDs) and Tomato Analyzer (TA) descriptors.

## 2. Results

### 2.1. Field Evaluation Using Conventional Descriptors

#### 2.1.1. Phenotyping of Vegetative and Reproductive Traits

Morphological traits were phenotyped at different growth stages and characterized using 28 traits related to vegetative and reproductive variations. Characterized morphological descriptors displayed a broad range of phenotypic variation among evaluated accessions (Table 1).

##### Vegetative Traits

The indeterminate accessions (59.3%) dominated compared to determinate, semi-determinate, and dwarf accessions, however 61 accessions belonging to the latter three growth habits showed 40.7% variation (Table 1). Medium maturity for flowering was observed in 89.7% of the accessions, while early and late flowering accessions displayed 11.3% variation (Table 1). Most early flowering accessions belonged to cherry and a processing salad type while late flowering accessions were variably distributed across all varietal groups. Most of the accessions from *S. lycopersicum* showed standard, dwarf, and potato leaves except the peruvianum leaf type seen in *S. peruvianum* accessions LYS-26 and LYS-33.

##### Fruit Color and Size

Fruit color in 94% of accessions showed green fruits when the fruits were immature. At the mature stage, fruits transitioned to an array of colors with the majority of accessions having red fruits. A total of 81 accessions with fruit colors ranging from yellow, orange, pink, orange red, or brown exhibited 54% variation (Table 1). Wild accessions LYS-26 and 33 belonging to peruvianum complex were still green in color when the fruits reached maturity.

Appreciable fruit size diversity was reported with most accessions having medium fruits. However, accessions with variable sizes ranging from small to large fruits exhibited 66% variation (Table 1). Fruit size variation was moderate, with one-third of accessions showing uniform fruit size, while the remaining accessions displayed slight to medium fruit size variation.

##### Traits Related to Fruit Form

Fruit texture measured by fruit fasciation was mostly smooth and flat-shaped at the blossom end. In 70% of accessions, no rib was observed at the calyx end, while the remaining accessions exhibited 30.7% variation for slight to medium ribs (Table 1).

Conventionally, varietal grouping of the tomato is established based on end use, but fruit shape also appears to be an important descriptor that can be used to establish varietal groupings. Hence, we utilized fruit shape to classify accessions into nine distinct varietal groups with most accessions belonging to rounded (33%), slightly flattened (23%), and high rounded (22%) shapes. Accessions belonging to varietal groups other than rounded shape displayed considerable fruit shape variation (Table 1).

##### Fruit Yield and Productivity

Fruit yield per plant allowed us to identify the yield potential of all evaluated accessions. More than 50% of accessions were moderately yielding, and nearly 20% were high yielding (Table 1). These accessions can be further utilized in the development of high-yield breeding lines. Fruit weight ranged from 1.23 g (LYS-33, peruvianum species) to 576.6 g (LYS-5) as shown in Table 2. Regarding productivity, moderate productivity was seen at an average of 1858.5 g, with LYS-33 (99.7 g) as the least productive and LYS-37 (5888 g) as the most productive.

### 2.2. TA Descriptor Characterization

In addition to field evaluation, fruit diversity was also comprehensively studied by TA fruit descriptors of fruit size, shape, and color.

#### 2.2.1. Fruit Trait Variation between and within Varietal Groups

**Figure 1:**
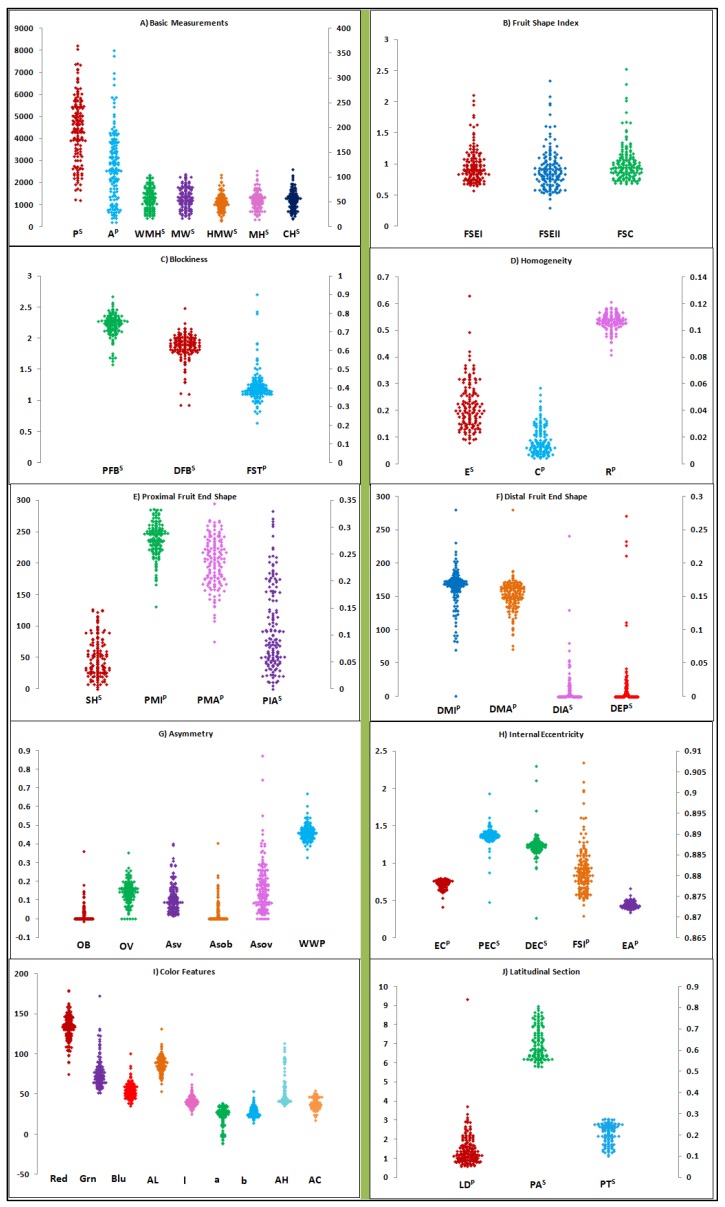
Tomato Analyzer (TA) fruit morphometric and colorimetric descriptor variation illustrated by Scattergram. External fruit features from longitudinal section are measured using basic measurements (**A**), shape (**B**–**H**), and color (**I**) descriptors. Internal fruit features of pericarp, placenta, and septum are measured using the latitudinal section (**J**). Descriptors with superscripts P and S are plotted with reference to the primary (left) and secondary axis (right), respectively. Scattergrams are explained by three axes (X, Y, and Z) with X axis displaying a descriptor for the given descriptor category, whereas Y and Z axis explain the primary (P) and secondary axes (S), respectively.

The Tomato Analyzer (TA) revealed intricacies associated with external and internal fruit features. Longitudinal fruit sections were able to generate comprehensive information about fruit size (Figure 1A), shape (Figure 1B–H), and color features (Figure 1I). Information related to the internal features of the pericarp, placenta, and septum were difficult to extrapolate by longitudinal section; hence latitudinal fruit sections were utilized to detect the accession variation of the pericarp area and thickness (Figure 1J). Across varietal groups, all 47 TA descriptors demonstrated significant variation for different fruit morphometric and colorimetric traits (Table 2). The highest range of variation was observed for fruit homogeneity, asymmetry, and proximal/distal fruit end (Table 2). Among all TA descriptors, proximal eccentricity and distal eccentricity descriptors showed values of 0.32 and 0.39, respectively, and least variation was obtained. Within each varietal group, variation for more than 32 TA descriptors was observed to be highly significant for slightly flattened, rounded, high rounded, cylindrical, and pyriform varietal groups (Table S1). The varietal groups of flattened, ellipsoid, and heart shape displayed significant differences for 19, 0, and 18 fruit descriptors, respectively. Within varietal groups, TA descriptors related to fruit blockiness, homogeneity, fruit end shape, asymmetry, and internal eccentricity did not show significant differences for the flattened and heart shape varietal groups (Table S1).

#### 2.2.2. Cluster Analysis

**Figure 2:**
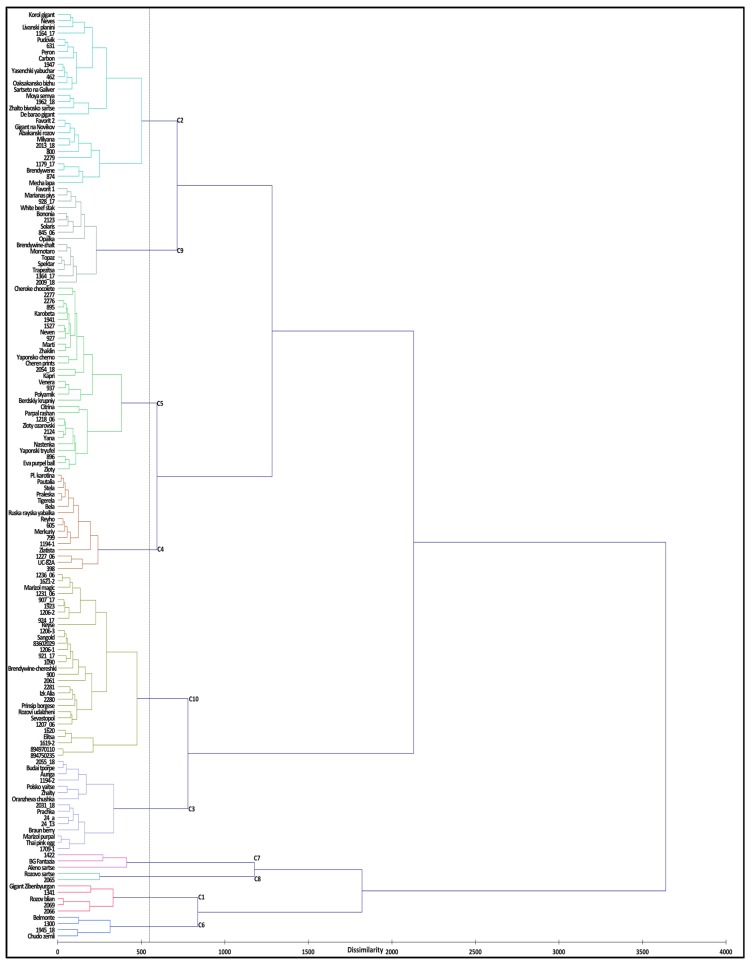
Hierarchical cluster analysis based on morphometric and colorimetric TA descriptors for evaluated tomato accessions. Euclidean Ward’s method for dissimilarity was utilized during agglomerative hierarchical clustering.

Cluster analysis was utilized to identify distinct clusters based on germplasm classification patterns. Overall, 47 TA descriptors (Table S2) were used for agglomerative hierarchical clustering with Ward’s coefficient function and 150 accessions were classified into 10 different clusters (Figure 2). Variance within class was 1.90% and between classes was 98.10%. Most accessions were populated into clusters 5 and 10 followed by clusters 2, 9, 4, and 3. Cluster 5 was mainly populated by accessions of medium size fruits, high round to round shape (Figure 2 and Table S3) with red, pink, orange, and brown fruits. Accessions belonging to cluster 10 were characterized by small fruit size, rounded and high rounded shape, in red, yellow, and orange colors. Cluster 2 represented big fruit accessions with slightly flattened shape, colored red or pink. Clusters 9 and 4 were also populated with accessions with big size fruits, flattened shape, and red colored. Accessions from clusters 1, 6, 7, and 8 were characterized by very big fruit size, flattened shape, colored red or pink (Figure 2 and Table S3). In regard to fruit color, accessions spread across different clusters were mostly populated with red and pink colored fruits; however, some accessions within each cluster had fruits colored orange, yellow, brown, and a mix of different color shades (Table S3). Regarding geographical distribution, accessions belonging to different countries were spread into different clades regardless of their shape and size (Table S3). Mostly, accessions from Bulgaria, Russia, USA, and China were represented in almost every cluster. Single accessions were observed in cluster 2, originating from Lebanon, Argentina, and Mexico; in cluster 3 from Hungary and Netherlands; and in clusters 4 and 9, from Belarus and Czech Republic, respectively.

#### 2.2.3. Multivariate Analysis

**Figure 3:**
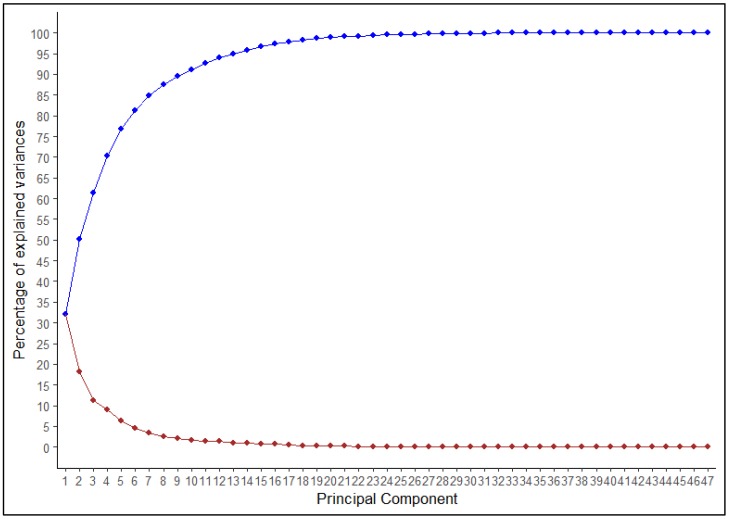
Principal component analysis (PCA) variance plot illustrating percent variation explained by each principle component. The blue line indicates the cumulative variation of 1–47 principal components, whereas the red line indicates variation explained by each individual component.

Multivariate analysis is an effective tool to quantify divergence among populations due to variable traits. The 47 TA descriptors were further analyzed by factor analysis to identify strongly correlated descriptor features (Table S2). Factor analysis revealed eight major principal components that contributed to the majority of the total cumulative variance. The proportion of each descriptor’s variance concerning the extracted factors is illustrated by descriptor communality (Table S2). Most variables (45 TA descriptors) had high commonality (>0.50) with the exception of distal angle micro and proximal eccentricity. PC1 to PC8 contributed 32.0%, 18.1%, 11.2%, 9.0%, 6.4%, 4.7%, 3.5%, and 2.6% variation, respectively, a total of 87.5% of the variance (Figure 3).

**Figure 4:**
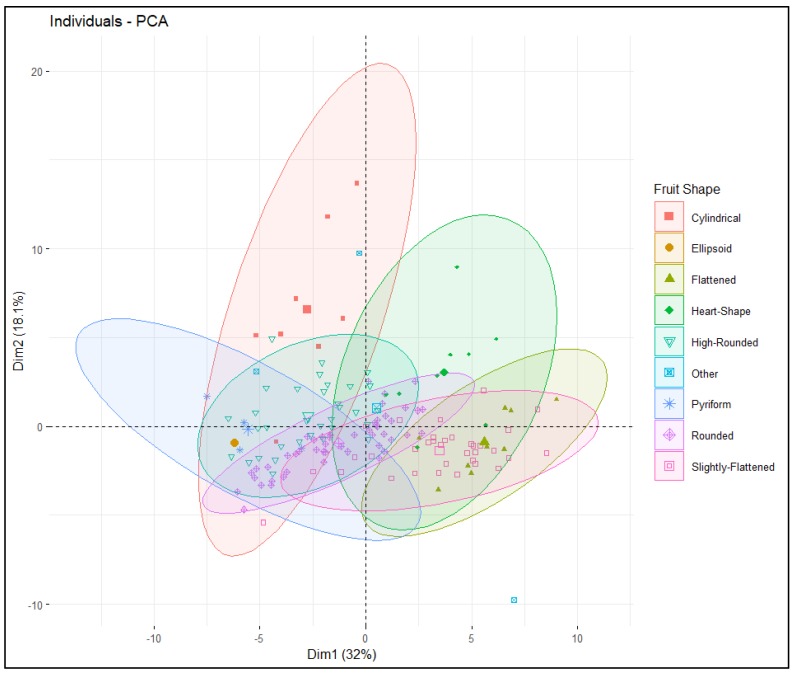
PCA ellipse plot displaying clusters of tomato accessions categorized by fruit shape. Each ellipse represents accessions with specific fruit shape, and different symbols and colors are assigned to display different fruit shape groupings.

**Figure 5:**
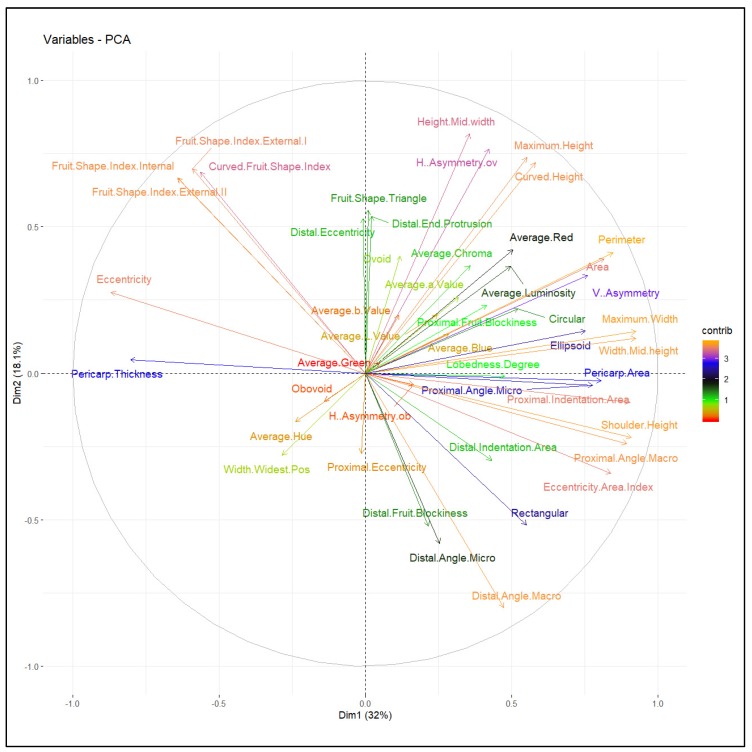
PCA feature plot displaying fruit morphometric and colorimetric descriptors.

Intra- and intervarietal group variation explained by PC1 and PC2 was 32% and 18.1% respectively, for a total of 50.1% (Figure 4). All tomato accessions were dispersed across all quadrants of the principal component analysis (PCA) ellipse plot and displayed no distinct clustering (Figure 4); however, accessions belonging to specific varietal groups did mostly populate specific quadrants based on TA descriptors (Figure 5). Accessions belonging to heart shape were limited to the positive quadrant of PC1 and PC2, while most accessions from the cylindrical and pyriform shape were found in the negative quadrant of PC1 and positive quadrant of PC2. Accessions belonging to rounded and high rounded shape were spread across all four quadrants (Figure 4). Basic measurements contributed more highly to PC1, whereas proximal and distal fruit end shape, and fruit shape index internal descriptors contributed to PC2 (Figure 5 and Table 3). In PC1 perimeter, area, width-maximum height, maximum width, shoulder height, proximal angle, and indentation area contributed positively whereas fruit shape index external, curved fruit shape index, distal fruit blockiness, and fruit shape triangle contributed negatively (Figure 5). In PC2 height mid-width, maximum width, and curved height, fruit shape index, and blockiness descriptors except proximal fruit blockiness explained positive variance while proximal/distal fruit end shape contributed negatively except distal end protrusion (Figure 5). Related TA descriptors were mostly found in the same quadrant whereas dissimilar descriptors were found in the opposite quadrants (Figure 5). Basic measurements related to fruit size and color descriptors were dispersed in the positive quadrant of PC1 and PC2; accessions found in this quadrant are ideal for breeding fruits for desirable size and dark red color. Obovoid and width widest position descriptors of fruit asymmetry, as well as average hue of color features explain the variation in the negative quadrant of PC1 and PC2 (Figure 5) and most slightly flattened accessions were populated in this quadrant.

#### 2.2.4. Correlation Network

**Figure 6:**
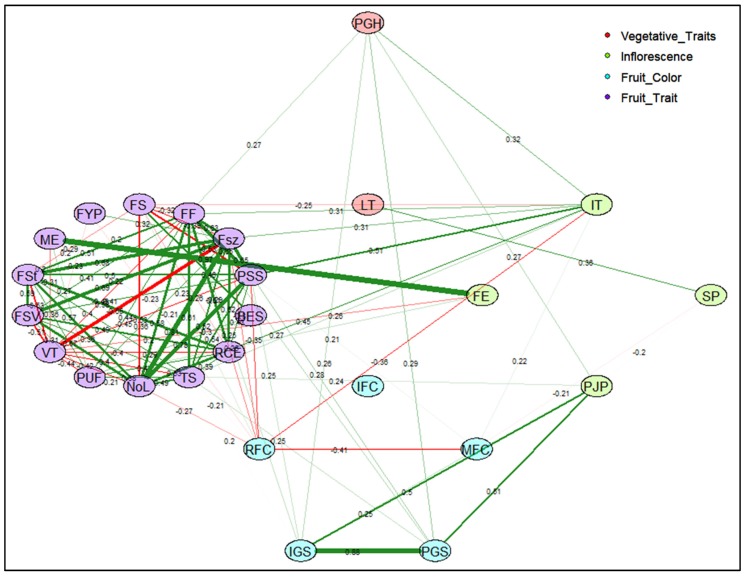
Correlation network for conventional descriptors (CD) illustrates the relationships between vegetative and fruit traits. Abbreviations used for morphological traits are represented as shown here. PGH: Plant Growth Habit; LT: Leaf Type; IT: Inflorescence Type; FE: Flowering Earliness; SP: Style Position; PJP: Presence of Jointless Pedicel; IFC: Immature Fruit Color; RFC: Ripened Fruit Skin Color; MFC: Mature Fruit Color; PGS: Presence of Green Shoulder; IGS: Intensity of Green Shoulder; RCE: Ribbing at Calyx End; TS: Transverse Section; NoL: Number of Locules; PUF: Puffiness; VT: Varietal Types; FSV: Fruit Size Variation; FSt: Fruit Setting; ME: Maturity Earliness; FYP: Fruit Yield per Plant; FS: Fruit Shape; FF: Fruit Fasciation; FSz: Fruit Size; PSS: Pistil Scar Shape; BES: Blossom End Shape. The number shown across each band represent the correlation coefficient between CD descriptors. Width of each band represents the strength of correlation among CD descriptors and oval/ellipse of specific color represents descriptor belonging to assign CD descriptor category. Positive correlations are shown by green color bands and negative correlations are displayed by red color bands.

**Figure 7:**
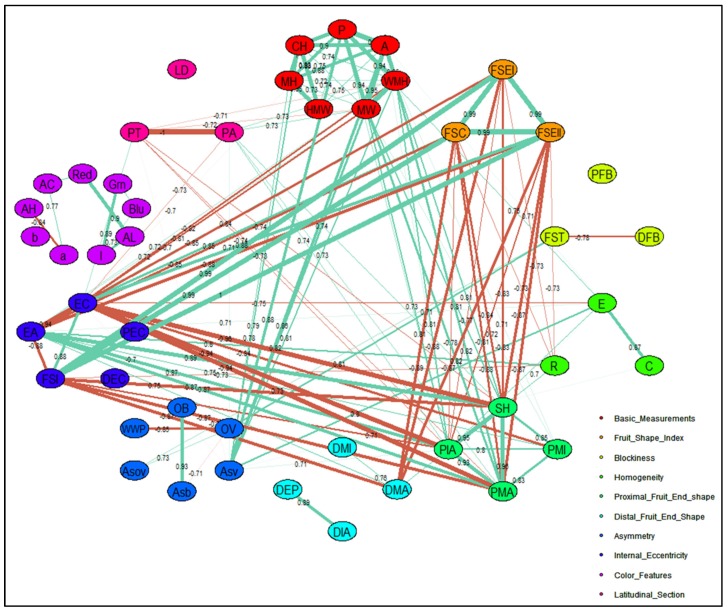
TA descriptor correlation network displaying relationship between fruit shape, size, and color descriptors. Abbreviations adapted for 47 TA descriptors are shown in Table 2 and a total of 10 descriptor categories are used to display the relationship between fruit shape, size, and color descriptors. The number shown across each band represent the correlation coefficient between TA descriptors. Width of each band represents the strength of correlation among TA descriptors and oval/ellipse color of specific color represents descriptor belonging to assign TA descriptor category. Positive correlations are shown by aquamarine color bands and negative correlations are displayed by coral color bands.

Correlations between traits were further investigated by a correlation matrix for CD (Figure S1A and Table S4) and TA descriptors (Figure S1B and Table S5). Furthermore, the relationship between strongly correlated and closely interacting descriptors for CD and TA descriptors were explained by respective correlation networks (Figure 6 and Figure 7). Correlations with an absolute value >0.1 and >0.7 were included to construct the correlation network for CD and TA descriptors, respectively. The width of each band represents correlation strength, whereas the colors grey and red illustrate the positive and negative correlations between descriptors, respectively. Most CDs were positively correlated except for varietal type and fruit size, fruit setting and fruit size variation, and ripened fruit skin color with mature fruit color (Figure 6). Flowering earliness and maturity earliness were in full synchrony and closely related. In regard to the relationship between TA descriptors, fruit size, shape, proximal fruit end, and internal eccentricity showed a close association (Figure 7). Fruit blockiness, distal fruit end, asymmetry, pericarp area, and thickness were negatively associated. Noticeably, color descriptors and latitudinal section traits related to pericarp were distinctly separated from those related to fruit shape and size.

Overall, multivariate analysis gave insight into the separation of different varietal groups. Multidimensional visualization of fruit shape, size, and color traits showed the contribution of individual descriptors to total variation. Comparative correlation networks between CD and TA descriptors allowed assessment of phenotypic diversity expressed at pre- and post-harvest stages, and it was observed that the diversity explained by fruit TA descriptors was higher than for CD. Correlations observed between different TA descriptors were highly variable compared to correlations between CDs.

## 3. Discussion

The key question remains of how variation in morphometric descriptors impacts overall phenotypic diversity related to vegetative and fruit traits. Phenotypic diversity of vegetative and yield related traits are studied extensively, but characterization of quantitatively inherited fruit shape and size is still limited [36]. In the present work we examined the morphological diversity of a tomato collection, representing the major fruit shape-based varietal groups, during pre- and post-harvest stages. By examining vegetative and fruit descriptors, we assessed inter- and intra-population variability represented by different varietal groups. The phenotypic diversity findings of this research support the proposed hypothesis and are in accordance with previously published works on tomato genetic diversity [1,28,37,38].

As anticipated, the present tomato collection displayed broad diversity in plant and fruit traits as given by CD and TA phenotypic descriptors. This suggests that appreciable genetic diversity for plant architecture, inflorescence, and fruit traits is present in this collection. The Balkan accessions were characterized with low morphological variability in comparison to accessions introduced from other geographical regions. Similar differences in morphological variability were observed by Mohan et al. between tomato accessions from India and accessions derived from the Tomato Genetics Resource Center (TGRC) [1]. Figàs et al. [28] postulated that a broad range of variation observed for fruit traits could encourage local production of tomatoes for different uses, and the large differences in highly specific fruit traits observed in this collection would be useful in establishing a tomato core collection. Based on monomorphic descriptors, cultivated accessions distinguished from wild species (LYS 26 and 33) were consistent with Díez and Nuez [26] and Figàs et al. [28].

Genetic diversity as measured by fruit TA descriptors appeared higher than for CDs related to plant, flower, and fruit morphology. Differences in the two assessment approaches as well as the quantitative nature of fruit shape and size likely explain the increased diversity observed for fruit features. The diversity of TA phenotypic descriptors likely reflects control by a large number of loci, as most descriptors related to fruit size and shape are polygenic in nature [38,39,40]. However, this needs to be further validated by molecular characterization, which was not within the scope of this study [28,37,41,42].

Different studies in the past have asserted that selection among local cultivars with a varietal group can improve yield and quality [29,43,44]. Fruit morphology is an essential criterion for establishment of different varietal groups [26,37,45], and our collection was distinctly categorized into nine varietal groups based on fruit shape and size similar to a collection of 127 tomato accessions studied by Mohan et al. [1]. In contrast, a collection of 58 Italian tomato accessions displayed only six varietal groups [46]. However, the Mercati et al. study was limited to long-term storage tomato accessions, hence total varietal groups may have been fewer than reported in this study. The genetic diversity reported for TA descriptors was variable across newly established varietal groups, illustrating low intra-varietal variation yet revealing large intervarietal variation. Genetic variability found for specific fruit shape, size, and color among inter- and within intravarietal groups would open up ways to select elite cultivars from the studied collection, as previously done by Greek [17], Italian [37], and Spanish [47] tomato researchers.

Distinctness analysis of plant and fruit diversity was carried out in tomato [1,17,28,46], pepper [35,48,49], and eggplant [50]. Distinctness, as identified using hierarchical clustering, multivariate and correlation network analyses, explains the impact of fruit shape and size descriptors on overall fruit diversity, and the findings of our study report similar observations, with the exception of clear separation of the varietal groups tested here. We anticipated that different varietal groups would be distinctly separated based on TA descriptors, but instead they overlapped together. This is in contrast to Figàs et al.’s [28] findings, but in agreement with the results of Cebolla-Cornejo [51], which were based on morphological fruit descriptors. The studied collection included fruits of varying sizes ranging from a small cherry to the largest heart shape, and so the observed variation might have confounded with other descriptors preventing distinct separation of varietal groups. In addition to fruit shape and size associated morphometric traits, fruit weight or mass is highly associated with pericarp, placenta, lobedness area, and locule numbers. As these traits are positively correlated with fruit mass and size, understanding them is important for characterization of the genetic basis of these phenotypes [52]. In addition to fruit morphometric and colorimetric trait characterization, TA was utilized to find quantitative trait loci (QTL) related to fruit shape and size in tomatoes [42,52] and peppers [48,53,54,55]. We intend to use data on fruit shape, size, and color traits reported here for subsequent QTL identification and validation using genome wide association studies (GWAS) and, ultimately, introgression into elite breeding lines in our tomato breeding program. Moreover, obtained detailed description of the tomato germplasm will help in the creation of a tomato core collection and future breeding program in selecting lines to develop high yielding F_1_ hybrids.

In this study we sought to investigate the use of a combined implementation of CDs and TA descriptors to measure the relationship among varietal groups of a tomato collection. Accessions collected and introduced from the same country or geographical region were spread and distributed across different clades and varietal groups, suggesting that each region is associated with its own diverse cultivars, as reported in a Turkish-Iranian tomato genetic diversity study [25]. These genetically distant accessions could be further used to broaden genetic variation and establish a core collection that showcases Bulgarian tomato diversity, which may be valuable for conservation and the utilization of local and introduced genetic resources.

## 4. Materials and Methods

### 4.1. Plant Material

**Figure 8:**
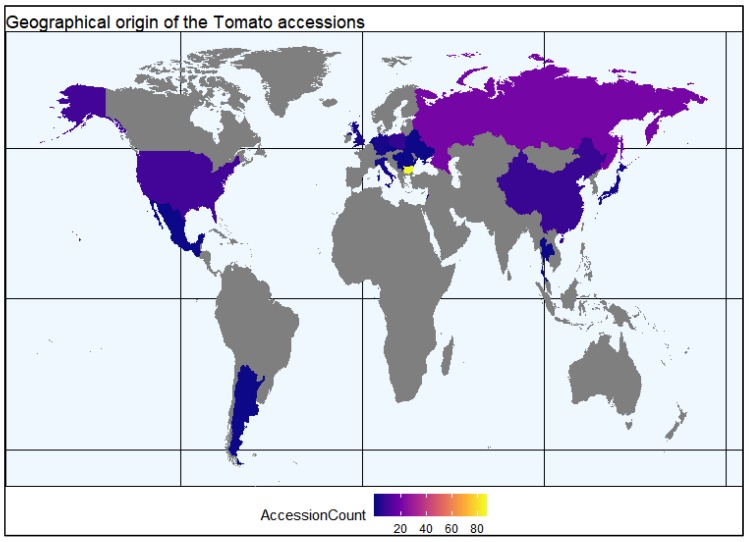
Geographical origin of the tomato accessions studied. Argentina (1), Belarus (1), Bulgaria (85), China (7), Czech Republic (1), Germany (2), Guatemala (1), Hungary (1), Italy (2), Japan (2), Lebanon (1), Mexico (1), Netherlands (1), Poland (6), Romania (1), Russia (20), Serbia (1), Thailand (1), UK (2), Ukraine (1), and USA (12).

**Figure 9:**
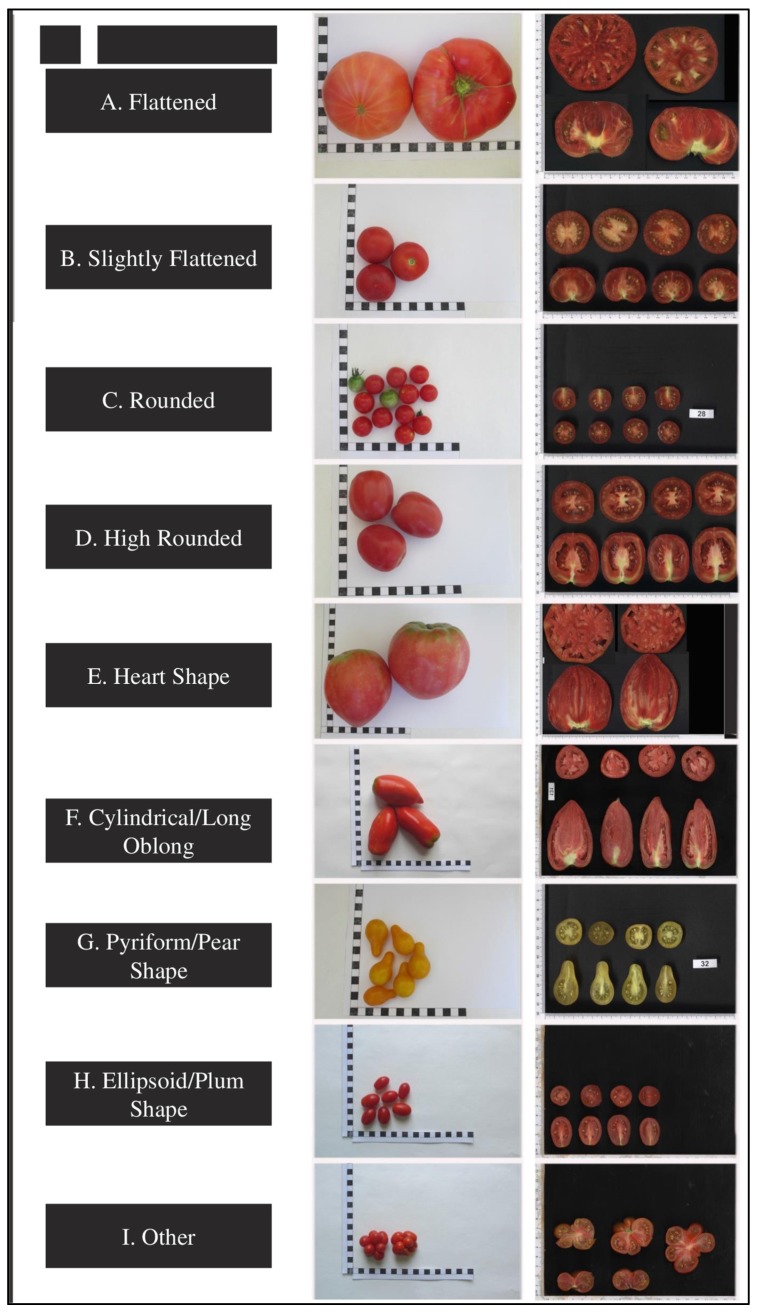
Representative fruit variability of different varietal types (column 1) displayed in whole fruit (column 2), longitudinal (column 3 bottom), and latitudinal fruit section (column 3 top).

A total of 150 tomato accessions representing 21 countries were included in this investigation. Geographical regions with number of accessions representing these regions are shown in Figure 8 and Table S6. Most tomato accessions belonged to *Solanum lycopersicum* L., except LYC-26 and LYC-33 accessions, which are part of *Solanum peruvianum* L. Accessions belonging to different fruit shape-based varietal groups are commonly recognized in tomato growing regions as shown in Figure 9. Based on fruit shapes, the evaluated tomato accessions were grouped into nine varietal groups of flattened, slightly flattened, rounded, high rounded, heart shape, cylindrical, pyriform, ellipsoid, and other fruit types.

### 4.2. Seed Germination, Transplanting, and Plant Growth

Each accession was represented by 10 plants in an open field plot trial with three replications in a randomized complete block design in Plovdiv, Bulgaria (GPS coordinates: 42°10′35.3″ N 24°45′50.5″ E) during the spring–summer season of 2018. Seeds were sown at the end of March in an unheated greenhouse and five-week-old tomato seedlings were transplanted in the field at the beginning of May. Plants were planted in a two-row planting scheme (110–50/25–30 cm) for determinate and one-rowed (80/30 cm) for indeterminate accessions.

### 4.3. Morphological Characterization

During different phenological growth stages, pre-harvest morphological characterization was mainly based on 28 conventional descriptors related to plant architecture, leaf, inflorescence, and fruit traits, whereas post-harvest fruit evaluation was based on 47 TA descriptors associated with fruit morphometric and colorimetric traits.

#### 4.3.1. Conventional Descriptor Characterization

Individual plants were characterized using 28 conventional descriptors [21]. These descriptors included plant architecture (7), inflorescence (3), and fruit (15) descriptors. Among the evaluated conventional descriptors, 7 traits were quantitatively measured whereas 21 traits were qualitatively assessed. Traits of the number of fruits per plant, fruit weight (gm), and productivity per plant (gm) were included to assess yield potential. The conventional descriptor data were collected from ten randomly selected plants or fruits from each of the three replications separately.

#### 4.3.2. Tomato Analyzer Descriptor Characterization

Eight fruits per accession from three different replications were analyzed by Tomato Analyzer for fruit morphometric [33,52,56] and colorimetric descriptors [34,57]. The fruit was scanned with longitudinal and latitudinal fruit section using Epson Perfection V19 J371A photo scanner (Epson, Amsterdam, The Netherlands) at a resolution of 300 dpi. Evaluated accessions were studied for 47 morphometric and colorimetric descriptors using TA version 3.0 software. Morphometric descriptors included basic measurements (7), fruit shape (3), blockiness (3), homogeneity (3), proximal/distal fruit end shape (4), asymmetry (6), and internal eccentricity (5), whereas colorimetric descriptors (8) included color features.

### 4.4. Statistical Analyses

Pre-harvest field data and large scale fruit image data were pre-processed and statistically analyzed using SAS, XLSTAT, and R program. The manuscript structure was inspired from previous work on tomato genetic diversity [1] and Balkan pepper fruit diversity [50] with inclusion of pre- and post-harvest vegetative and fruit traits comprehensive analysis.

#### 4.4.1. Construction of World Map

The world map representing geographical origin of evaluated tomato accessions was constructed using *ggplot* package of R program.

#### 4.4.2. Analysis of Variance (ANOVA)

Differences between groups, within each group, and among accessions were detected by adopting a general linear model (GLM) using SAS Version 9.2. (SAS Inst., Inc., Cary, NC, USA). ANOVA was performed on individual fruits (TA descriptors) to detect differences among accessions. The TA descriptors scattergrams were built using XLSTAT version 15.

#### 4.4.3. Hierarchical Cluster Analysis (HCA) and Factor Analysis

A total of 47 TA descriptors were used for clustering 150 tomato accessions using Ward’s coefficient by agglomerative hierarchical clustering in XLSTAT. Factors were analyzed by TA descriptors and factors with eigenvalues >1 were extracted by varimax rotation.

#### 4.4.4. Multivariate Analysis

Principal component analysis (PCA) was utilized to understand between and within-varietal groups variation. Different PCA parameters were estimated using *ggplot2*, *missMDA*, *FactoMineR,* and *Factoextra* R packages. In addition to PCA, a separate correlation coefficient heatmap and a correlation network were also estimated to understand how different conventional descriptors (CDs) and TA descriptors contributed to pre-harvest plant architectural and post-harvest fruit diversity. The correlation matrix between different CDs and TA descriptors were estimated using the *cor* function, whereas the coefficient heatmap was generated using *ggcorrplot* and the correlation network was constructed using *qgraph*.

## 5. Conclusions

A combined approach using conventional and TA descriptors was undertaken to study the tomato plant, inflorescence, and fruit diversity. This approach allowed us to detect variation for vegetative and fruit traits among evaluated local forms and breeding lines, and introduced tomato varieties. In addition to CDs of plant morphological traits, our findings demonstrate that the TA and data visualization tools were immensely useful in discerning associations between fruit shape, size, and color descriptors. Overall, collecting phenotypic variation data enabled us to index a collection of Balkan tomatoes and introduced tomato accessions into fruit shape-derived varietal groups. The results of this investigation were concomitant with previous tomato genetic diversity studies of vegetative (CD) and fruit descriptors (TA). The variation reported for CD and TA quantified fruit shape, size, and color descriptors will be used to establish a tomato core collection and further exploited to select and breed a desirable fruit shape tomato. This data will also be useful for an investigation into the genetic determinants of polygenic traits using GWAS.

## Figures and Tables

**Table 1:** Phenotypic variation of representative vegetative and fruit traits and their percentage variation in the population.

		Category	Sub-Category	No. of Accessions	Accessions Showing Variation	% Variation
**I**	**Architecture**	**1. Plant growth habit**	Dwarf	4	61	40.7
			Determinate	36		
			Semi-Determinate	21		
			Indeterminate	89		
**II**	**Leaf**	**2. Leaf type**	Dwarf	4	17	11.3
			Potato Type	11		
			Standard	133		
			Peruvianum	2		
**III**	**Inflorescence**	**3. Inflorescence Type**	Uniparous	70	80	53.3
			Both	40		
			Multiparous	40		
		**4. Style Position**	Inserted	0	2	1.3
			Same as Stamen	148		
			Slightly Exerted	2		
			Highly Exerted	0		
		**5. Presence of Jointless Pedicel**	Absent	37	37	24.7
			Present	113		
		**6. Flowering Earliness**	Early	8	15	10
			Medium	135		
			Late	7		
**IV**	**Fruit**	**7. Fruit size**	Very Small	18	99	66
			Small	24		
			Medium	51		
			Large	40		
			Very Large	17		
		**8. Fruit Shape**	Flattened	9	101	67.3
			Slightly Flattened	34		
			Rounded	49		
			High Rounded	33		
			Heart Shape	9		
			Cylindrical	8		
			Pyriform	4		
			Ellipsoid	1		
			Other	3		
		**9. Immature Fruit color**	Greenish White	2	10	7.0
			Light Green	7		
			Green	140		
			Dark Green	1		
		**10. Mature Fruit color**	Green	2	81	54
			Yellow	8		
			Orange	18		
			Red	69		
			Pink	34		
			Orange Red	3		
			Brown	8		
			Other	8		
		**11. Ripened Fruit Skin Color**	Colorless	37	37	24.7
			Yellow	113		
		**12. Presence of Greenback**	Present	54	54	36
			Absent	96		
		**13. Intensity of Greenback**	Absent	54	92	61.3
			Slight	25		
			Intermediate	58		
			Strong	13		
		**14. Fruit Size Variation**	Uniform	53	86	57.3
			Slight	64		
			Medium	32		
			High	1		
		**15. Fruit Setting**	Low	91	91	60.7
			Intermediate	52		
			High	7		
			Very High	0		
		**16. Varietal Type**	Salad	18	80	53.3
			Beef	9		
			Roma	20		
			Processing Salad	70		
			Cherry	32		
			Pear	1		
		**17. Fruit Yield per Plant**	Very Low	11	70	46.7
			Low	31		
			Medium	80		
			High	25		
			Very High	3		
		**18. Ribbing at Calix End**	Absent	104	46	30.7
			Slight	12		
			Medium	32		
			Strong	2		
		**19. Blossom End Shape**	Flat	125	25	16.7
			Indented	24		
			Unknown	1		
		**20. Maturity Earliness**	Early	8	14	9.0
			Medium	136		
			Late	6		

**Table 2:** Descriptive statistics and analysis of variance (ANOVA) for yield components (A) and TA descriptors (B). Level of significance expressed is nonsignificant (NS) *p* > 0.05, * *p* < 0.05, ** *p* < 0.01; *** *p* < 0.001. ^#^ TA descriptor codes are adapted from Tripodi and Greco [35] pepper study.

Descriptor (Unit)	Code ^#^	Across Varietal Types					
		Descriptive Statistics			ANOVA		
					F-Value	Sum of Squares (%)	
		Mean	Range	CV	Accession	Accession	Residual
Fruit Weight (gm)		132.4	1.23–576.6	33.7	70.25 ***	206,808	26,695
Fruits per Plant		29.9	4.0–267.7	25.4	67.30 ***	5802	150
Productivity (gm)		1858.5	99.7–5888.0	34.8	7.23 ***	4,517,679	1,169,403
**Basic Measurements:**							
Perimeter (mm)	P	191.91	53.52–365.8	9.79	43.34 ***	22,821.37	1374.82
Area (mm^2^)	A	2617.43	202.1–8002.9	19.54	32.50 ***	12,670,602	1,017,690
Width Mid-Height (mm)	WMH	56.05	16.87–104.7	11.69	38.13 ***	2443.20	167.26
Maximum Width (mm)	MW	56.63	16.9–105.4	11.56	38.63 ***	2466.26	166.68
Height mid-Width (mm)	HMW	48,11	11.5–105.2	8.28	53.07 ***	1255.66	61.767
Maximum Height (mm)	MH	53.15	14.1–112.6	7.99	58.27 ***	1567.98	70.251
Curved Height (mm)	CH	54.41	16.2–115.7	8.63	48.03 ***	1578.42	85.8002
**Fruit Shape Index:**							
Fruit Shape Index External I	FSEI	0.995	0.57–2.11	8.25	40.58 ***	0.4073	0.0262
Fruit Shape Index External II	FSEII	0.932	0.30–2.34	10.81	36.63 ***	0.5544	0.0395
Curved Fruit Shape Index	FSC	1.04	0.69–2.53	9.36	34.86 ***	0.4885	0.0365
**Blockiness:**							
Proximal Fruit Blockiness	PFB	0.737	0.525–0.890	7.51	4.06 ***	0.0186	0.0119
Distal Fruit Blockiness	DFB	0.619	0.309–0.826	8.78	6.72 ***	0.0296	0.0115
Fruit Shape Triangle	FST	1.23	0.63–2.70	15.51	6.76 ***	0.3662	0.1413
**Homogeneity:**							
Ellipsoid	E	0.042	0.016–0.126	20.55	12.97 ***	0.00145	0.00029
Circular	C	0.084	0.023–0.285	27.54	16.52 ***	0.0134	0.00212
Rectangular	R	0.534	0.408–0.607	4.51	5.88 ***	0.0051	0.00225
**Proximal Fruit End Shape:**							
Shoulder Height	SH	0.057	0.0–0.15	32.99	12.92 ***	0.00676	0.00137
Proximal Angle Micro (Degrees)	PMI	235.98	130.9–285.8	8.83	5.73 ***	3707.77	1689.38
Proximal Angle Macro (Degrees)	PMA	198.18	75.6–295.2	8.67	17.24 ***	7592.9	1149.96
Proximal Indentation Area	PIA	0.107	0.0–0.33	45.53	8.73 ***	0.0309	0.0093
**Distal Fruit End Shape:**							
Distal Angle Micro (Degrees)	DMI	163.46	0.50–280.2	15.44	4.15 ***	3944.70	2480.03
Distal Angle Macro (Degrees)	DMA	150.33	70.53–280.4	7.75	12.58 ***	2541.33	527.51
Distal Indentation Area	DIA	0.007	0.0- 0.24	265.85	4.28 ***	0.0022	0.0014
Distal End Protrusion	DEP	0.013	0.0–0.27	271.32	4.72 ***	0.0086	0.0048
**Asymmetry:**							
Obovoid	OB	0.02	−0.02–0.36	180.65	5.54 ***	0.0108	0.0051
Ovoid	OV	0.141	0.0–0.35	42.06	3.57 ***	0.0187	0.0137
V. Asymmetry	Asv	0.101	0.012–0.399	57.98	5.23 ***	0.0269	0.0134
H. Asymmetry. Ob	Asob	0.024	0.0- 0.41	231.6	3.47 ***	0.0161	0.0121
H. Asymmetry. Ov	Asov	0.166	0.00–0.87	53.83	7.62 ***	0.0909	0.0311
Width Widest Pos	WWP	0.461	0.33–0.67	8.97	3.51 ***	0.0089	0.0066
**Internal Eccentricity:**							
Eccentricity	EC	0.728	0.414–0.796	3.74	12.80 ***	0.0141	0.0029
Proximal Eccentricity	PEC	0.889	0.87–0.90	0.32	1.71 ***	0.00002	0.00003
Distal Eccentricity	DEC	0.887	0.87–0.91	0.39	2.19 ***	0.00004	0.00005
Fruit Shape Index Internal	FSI	0.933	0.29–2.34	10.85	36.56 ***	0.5577	0.0393
Eccentricity Area Index	EA	0.437	0.34–0.66	5.22	11.02 ***	0.0085	0.00202
**Average Color Values:**							
Red	Red	133.16	74.99–179.3	4.89	21.15 ***	1341.43	165.61
Green	Green	75.91	51.49–172.4	8.42	28.60 ***	1742.58	159.09
Blue	Blue	54.31	35.62–100.37	8.67	13.84 ***	456.65	86.14
Luminosity	AL	88.26	53.26–131.46	5.50	15.26 ***	535.91	91.67
L	l	41.47	35.58–35.71	6.07	21.71 ***	205.05	24.66
a	a	23.81	−11.7–38.85	11.46	58.79 ***	652.03	28.95
B	b	27.58	13.6–53.49	6.12	40.54 ***	172.03	11.08
Hue	AH	50.59	35.2–113.2	6.89	86.77 ***	1572.6	47.31
Chroma	AC	37.95	17.1–54.7	6.67	24.01 ***	229.4	24.95
**Latitudinal Section:**							
Lobedness Degree	LD	1.43	1.68–0.93	42.93	5.47 ***	3.055	1.357
Pericarp Area	PA	0.62	0.52–0.81	4.11	23.3 ***	0.022	0.0023
Pericarp Thickness	PT	0.21	0.10–0.28	7.39	23.1 ***	0.0086	0.0009

**Table 3:** Factor analysis-based relationship between different TA variables and factors.

TA Descriptor Category	Factor	Correlation between the Factor and Variable	
		Positive	Negative
**Size**	1	Basic measurement (size)	Eccentricity
**Shape**	2	Fruit shape index external I	Proximal/distal fruit end shape
		Fruit shape index external II	Fruit shape index internal
		Curved fruit shape index	
		H. asymmetry. ov	
	5	Average chroma	Fruit shape triangle
			Circular
	6	Pericarp area	Average chroma
	7		Proximal fruit blockiness
		Lobedness degree	Distal fruit blockiness
			Rectangular
	8	Height mid-width	Distal end protrusion
**Color**	3	Green	a
		l	
	4	a	Green
			l

## Supplementary Materials

The following are available online at https://www.mdpi.com/2223-7747/9/2/197/s1, Table S1: Descriptive statistics and analysis of variance (ANOVA) for within fruit shape-based varietal groups, Table S2: Proportion of variance explained by the extracted factors for each variable. Numbers 1–35, 36–44, and 45–47 represent shape, color, and pericarp features measured by TA, Table S3: Cluster analysis based class-wise accession description based on fruit shape, size, and color, Table S4: Correlation matrix of conventional descriptors (CDs), upper triangle represents *p*-values, whereas lower triangle represents correlation (R^2^) between CDs, Table S5: Correlation matrix of TA descriptors, upper triangle represents *p*-values, whereas lower triangle represents correlation (R^2^) between TA descriptors, Table S6: Passport data of the tomato accessions evaluated in this study including country of origin, population type, fruit shape, size, and color, Figure S1A: Correlation matrix heatmap of vegetative and fruit morphological descriptors, Figure S1B: Correlation matrix heatmap of TA descriptors.
