# Supplementary material for: Tomato Phenotypic Diversity Determined by Combined Approaches of Conventional and High-Throughput Tomato Analyzer Phenotyping
Source: Plants (Basel). 2020 Feb 5;9(2):197. doi: 10.3390/plants9020197 (PMC7076427; doi:10.3390/plants9020197)
Supplement: Supplementary file 1 [file plants-09-00197-s001.zip › Supplementary Information/Tables/Table S2 (TA Commanalities in Factor Analysis).docx]

**Table S3.** Proportion of variance explained by the extracted factors for each variable. Numbers 1-35, 36-44 and 45-47 represent shape, color and pericarp variables collected by TA.

| **Sr. No** | **Variables** | **Initial** | **Extraction** |  | **Sr. No** | **Variables** | **Initial** | **Extraction** |
| --- | --- | --- | --- | --- | --- | --- | --- | --- |
| **1.** | Perimeter | 1.000 | 0.920 |  | **25.** | Obovoid | 1.000 | 0.822 |
| **2.** | Area | 1.000 | 0.918 |  | **26.** | Ovoid | 1.000 | 0.838 |
| **3.** | Width Mid-height | 1.000 | 0.776 |  | **27.** | V Asymmetry | 1.000 | 0.707 |
| **4.** | Maximum Width | 1.000 | 0.789 |  | **28.** | H Asymmetry ob | 1.000 | 0.720 |
| **5.** | Height Mid-width | 1.000 | 0.908 |  | **29.** | H Asymmetry ov | 1.000 | 0.654 |
| **6.** | Maximum Height | 1.000 | 0.961 |  | **30.** | Width Widest Pos | 1.000 | 0.739 |
| **7.** | Curved Height | 1.000 | 0.963 |  | **31.** | Eccentricity | 1.000 | 0.682 |
| **8.** | Fruit Shape Index External I | 1.000 | 0.983 |  | **32.** | Proximal Eccentricity | 1.000 | 0.261 |
| **9.** | Fruit Shape Index External II | 1.000 | 0.991 |  | **33.** | Distal Eccentricity | 1.000 | 0.503 |
| **10.** | Curved fruit shape index | 1.000 | 0.985 |  | **34.** | Fruit Shape Index Internal | 1.000 | 0.991 |
| **11.** | Proximal fruit blockiness | 1.000 | 0.750 |  | **35.** | Eccentricity Area Index | 1.000 | 0.699 |
| **12.** | Distal fruit blockiness | 1.000 | 0.845 |  | **36.** | Avg Red | 1.000 | 0.844 |
| **13.** | Fruit shape Triangle | 1.000 | 0.744 |  | **37.** | Avg Green | 1.000 | 0.984 |
| **14.** | Ellipsoid | 1.000 | 0.828 |  | **38.** | Avg Blue | 1.000 | 0.758 |
| **15.** | Circular | 1.000 | 0.853 |  | **39.** | Avg Luminosity | 1.000 | 0.606 |
| **16.** | Rectangular | 1.000 | 0.615 |  | **40.** | Avg L | 1.000 | 0.873 |
| **17.** | Shoulder Height | 1.000 | 0.725 |  | **41.** | Avg a* | 1.000 | 0.812 |
| **18.** | Proximal Angle Micro | 1.000 | 0.564 |  | **42.** | Avg b* | 1.000 | 0.699 |
| **19.** | Proximal Angle Macro | 1.000 | 0.818 |  | **43.** | Avg Hue | 1.000 | 0.825 |
| **20.** | Proximal Indentation Area | 1.000 | 0.637 |  | **44.** | Avg Chroma | 1.000 | 0.885 |
| **21.** | Distal Angle Micro | 1.000 | 0.453 |  | **45.** | Lobedness Degree | 1.000 | 0.589 |
| **22.** | Distal Angle Macro | 1.000 | 0.767 |  | **46.** | Pericarp Area | 1.000 | 0.555 |
| **23.** | Distal Indentation Area | 1.000 | 0.637 |  | **47.** | Pericarp Thickness | 1.000 | 0.561 |
| **24.** | Distal End Protrusion | 1.000 | 0.588 |  |  |  |  |  |
