# Supplementary figures and images for: Tomato Phenotypic Diversity Determined by Combined Approaches of Conventional and High-Throughput Tomato Analyzer Phenotyping
Source: Plants (Basel). 2020 Feb 5;9(2):197. doi: 10.3390/plants9020197 (PMC7076427; doi:10.3390/plants9020197)

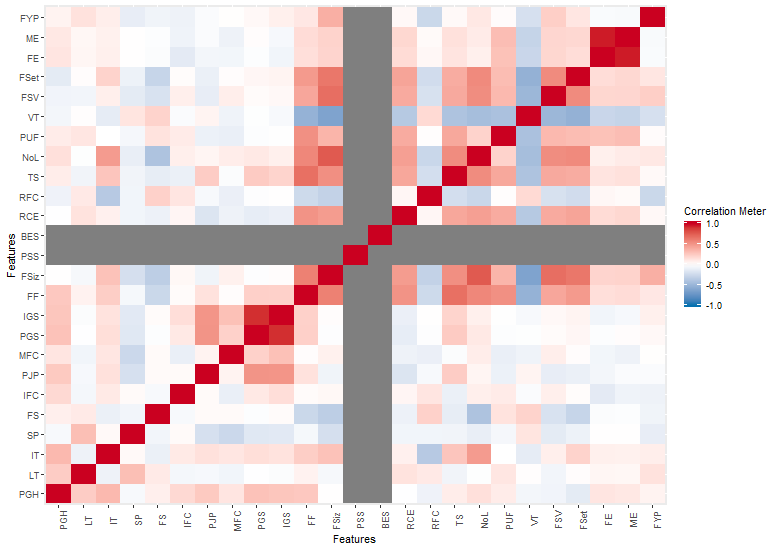

Supplement: Supplementary file 1 [file plants-09-00197-s001.zip › Supplementary Information/Figures/Fig S1A. Correlation Matrix Heatmap of CD Descriptors.png]

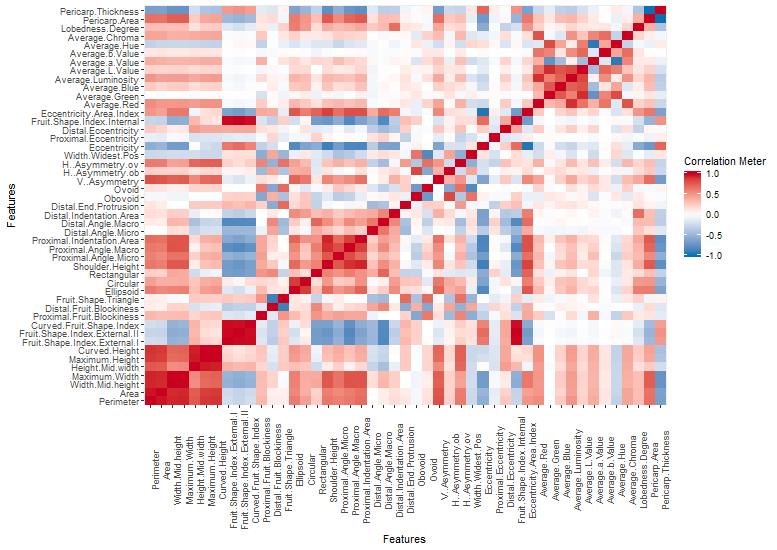

Supplement: Supplementary file 1 [file plants-09-00197-s001.zip › Supplementary Information/Figures/Fig S1B. Correlation Matrix Heatmap of TA Descriptors.png]
